# Photosynthesis under Environmental Fluctuations: A Challenge for Plants, a Challenge for Researchers

**DOI:** 10.3390/plants12244146

**Published:** 2023-12-13

**Authors:** Lorenzo Ferroni, Marek Živčak

**Affiliations:** 1 Department of Environmental and Prevention Sciences, University of Ferrara, 44121 Ferrara, Italy; 2 Institute of Plant and Environmental Sciences, Slovak University of Agriculture, 94976 Nitra, Slovakia

The ability of plants to cope successfully with environmental fluctuations is a result of their evolution in subaerial environments, where fluctuations in parameters such as temperature, light, and water availability, are the norm and stable states are the exception. Despite this, most studies have focused on plant responses to stable stress conditions, which are certainly more accessible for experimentation than fluctuating conditions. The concept of environmental fluctuation also includes a sense of *predictability*: some fluctuations are predictable, dictated, for example, by circadian rhythms, while others are unpredictable. Photosynthetic regulation, which takes place at all levels of organisation, from macroscopic morphology to the biochemical-molecular level, buffers the effects of fluctuations, limiting the over-reduction of the photosynthetic apparatus and the consequent production of reactive oxygen species. The latter are notorious for their detrimental effects on cell structures.

This Special Issue of *Plants* collects twelve high-quality scientific papers, and has additionally been supported by a large panel of expert reviewers who commented on the manuscripts. The papers offer a variety of viewpoints, reflecting the diverse scientific backgrounds of the authors. Moreover, due to the variety of plant species that were analysed in these studies, which are wide-ranging in both their systematic and environmental positions, this collection is able to present some interesting ecological and evolutionary insights.

The evolution of processes and structures is key to understanding the biological world and provides the context for plant responses to environmental fluctuations. Pushan Bag reviews and interprets the stages in the evolution of antenna systems in land plants, emphasising how the same antenna proteins can allow the optimisation of light harvesting in various light environments, which differ greatly in terms of light quality and quantity [1]. An extremely contemporary research topic is the possibility of increasing crop yields by downsizing the size of antennae [2]. However, this biotechnological operation may have side effects on the plant’s overall metabolism. Using a mini panel of wheat mutants with reduced chlorophyll content, Colpo et al. document that, in a fluctuating light environment such as the natural one, reduced antenna sizes not only impair the control of photosynthetic electron flow but also negatively influence plant morphogenesis, resulting in lower grain production [3]. Fluctuations in light intensity pose a significant challenge to photosynthetic regulation, resulting in a loss of carbon assimilation [4]. By exposing sunflower plants to pulsed light at different frequencies, Cinq-Mars and Samson conclude that the net photosynthesis loss at low frequencies (<5 Hz) depends on the shift from linear to cyclic electron transport around the PSI (CEF), leading to efficient photoprotection, but also a significant loss in carboxylation capacity [5].

More generally, the “time factor” is certainly decisive in dynamic photosynthesis, and the literature review by Li, Gao, and Zhang highlights the great diversity of photosynthetic behaviour among different species and cultivars [6]. In this regard, Wang et al.’s report is incredibly instructive, demonstrating how the selection of modern rose cultivars has led to increased efficiency in their use of fluctuating light, associated with higher levels of leaf stomatal conductance [7]. For a more comprehensive understanding of dynamic photosynthesis, Li et al. also list a number of methodological challenges and mention the importance of moving beyond the “single factor experiment” to consider a broader spectrum of environmental variables [6]. Taking the orchid species *Dendrobium officinale* as their example, Sun et al. provide evidence that the response to fluctuating light under high-temperature conditions requires, in addition to CEF activation, the water–water cycle to consume the electrons produced beyond the use capacity of photosynthesis in order to preserve the integrity of the PSI [8]. The importance of enhancing PSI protection under heat stress also emerges in the work presented by Filaček et al., who show that pre-acclimation to high temperature helps wheat develop a thermally resistant form of photosynthesis [9]. Their comparative experiment includes the low-chlorophyll mutant ANK-32A, which is known for its sensitivity to prolonged, moderate heat stress and could be particularly susceptible to repeated heatwaves [10]. However, the authors found that this is not the case [9], which suggests that plant responses to light and temperature fluctuations are definitely linked to each other but hardly predictable. The combined response to light and heat is the subject of the study proposed by Krevlaski et al.: using phytochrome mutants of the model angiosperm *Arabidopsis thaliana*, the authors offer evidence that a low red/far-red ratio is favourable for the prevention of PSII photoinhibition under moderate short-term thermal stress [11]. The stress-protective effect of red light is confirmed by Pashkovskiy et al., who report their experiment on the consequences of light quality manipulation on the secondary metabolism in *Pinus sylvestris* plantlets, observing effects on the quantity of antioxidants and carotenoids in needles [12].

Generally, high levels of antioxidants in leaves support an effective response to environmental fluctuations. Particularly intriguing is the case of the minute floating water plant *Lemna minor*, which is dealt with in the comprehensive review written by López-Pozo, Adams, Polutchko, and Demmig-Adams. The authors highlight a number of this duckweed’s unusual properties which allow it to thrive in a fluctuating environment [13]. Although this information may not be generalizable to other species, the environmental pressure of the aquatic environment, particularly the floating habitat, has certainly had a major role in the evolution of duckweed’s photosynthetic traits. Comparative studies of different species can further our understanding of their photosynthetic response to environmental fluctuations. This comparative approach was chosen by James Bunce to study the effect of fluctuating levels of CO_2_ supply. He discovered that, in four out of five analysed species, the fluctuations in CO_2_ decreased the carbon fixation due to lower PSII photochemical efficiency and stomatal conductance [14]. This finding is methodologically relevant to the simulation of higher atmospheric CO_2_ pressure scenarios, the effects of which have become increasingly evident with the rise of extreme weather events, ranging from severe drought to heavy rainfall, leading to soil flooding. Crops which are ideal for land particularly susceptible to recurrent extreme events should have sufficient photosynthetic flexibility to secure food despite such events. This problem is investigated by Chen et al., who exposed the tropical rainforest crop *Plukentia volubilis* to waterlogging stress and explored the possibility of selecting more tolerant lines based on their photosynthetic traits [15].

We are confident that our readers will appreciate all the papers in this collection for their scientific richness, variety of experimental approaches, and the diversity of the authors’ writing styles. Our hope is that they will be useful for the development of research on such a topical and fascinating subject as photosynthesis in an ever-changing environment.

## Data Availability

No new data were created or analyzed in this study. Data sharing is not applicable to this article.
